# SGA Children with Moderate Catch-Up Growth Are Showing the Impaired Insulin Secretion at the Age of 4

**DOI:** 10.1371/journal.pone.0100337

**Published:** 2014-06-30

**Authors:** Ivana Milovanovic, Falucar Njuieyon, Samia Deghmoun, Didier Chevenne, Claire Levy-Marchal, Jacques Beltrand

**Affiliations:** 1 INSERM CIE 05 – Unité d'épidémiologie clinique, Hôpital Robert Debré, Paris, France; 2 Service de biochimie et hormonologie, Hôpital Robert Debré, Paris, France; 3 Endocrinologie et diabétologie pédiatrique, Hôpital Necker, Paris, France; 4 Université Paris 5, René Descartes, Paris, France; 5 INSERM U845, Imagine Affiliated, Paris, France; Scientific Directorate, Bambino Hospital, Italy

## Abstract

**Background:**

Being born small for gestational age (SGA) is a risk factor for later development of type 2 diabetes. The development of glucose tolerance disorders in adults involves insulin resistance and impaired insulin secretion.

**Objective:**

To evaluate insulin secretion and insulin sensitivity in a 4-yr old cohort of SGA.

**Methods:**

85 children were prospectively followed from mid-gestation to 4 years of age. Fetal growth velocity (FGV) was measured using ultrasound measurements. Body composition and hormonal profile were measured at birth, 1 and 4 years.

**Results:**

23 SGA babies had lower birth weight compared to 62 AGA (−1.9±0.3 vs. −0.6±0.8 z-score; p<0.0001) and they were thinner at birth (ponderal index 24.8±1.8 vs. 26.3±3.1 kg/m3; p = 0.01 and fat mass 11±2.6 vs. 12.9±3.1%; p = 0.01). No significant differences in other measured metabolic and hormonal parameters were observed between two groups at birth. SGA infants experienced an early catch-up growth in weight (mean gain of 1.1±0.6 SD) during the first year of life. At 4 years, SGA children remain lighter than AGA, but with weight z-score in the normal range (−0.1±1.3 vs. 0.5±1.3 z-score; p = 0.05). No excess of fat mass was observed (19±4.8 vs. 19.7±4.1%; p = 0.45). 120-min plasma glucose was significantly higher (6.2±1.1 vs. 5.6±0.9 mmol/l; p = 0.006) and insulinogenic index was significantly lower (0.28±0.15 vs. 0.40±2.4; p = 0.02) in the SGA group at 4-yrs of life contrasting with a preserved insulin sensitivity (QUICKI 0.47±0.09 vs. 0.43±0.05; p = 0.06).

**Conclusion:**

SGA children with compensatory catch-up growth in first year of life show mild disturbances of glucose tolerance associated to a lower insulinogenic index at 4-yrs of age suggesting impairment of β-cell function.

## Introduction

The association between a low birth weight and development of type 2 diabetes has been consistently reported in numerous publications [1], [2] ; this association is strengthened after adjusting for adult BMI [3]. The pathway leading from small size at birth as a result of the exposure to an adverse fetal environment, to metabolic diseases later in life is not clear. The development of glucose tolerance disorders in adults involves insulin resistance and impaired insulin secretion. The early appearance of insulin resistance without obesity in SGA subjects is a well-known phenomenon. Thinness at birth (apart from birth weight itself) and the magnitude of the compensatory postnatal catch-up growth, which in turn induces abnormal growth of the adipose tissue, are two components that are associated with later insulin resistance [4], [5].

In animal models it has been demonstrated that in utero malnutrition affects pancreatic β-cell development leading to impaired β-cell function later in life [6], [7]. In contrast, insulin secretion in SGA born children has been poorly studied and yield conflicting results in adults [8]–[10]. Our understanding of fetal programming events in the human endocrine pancreas is then limited.

The aim of this study was to evaluate insulin secretion and insulin sensitivity in a cohort of SGA born children in whom both prenatal and postnatal growth have been monitored in a prospective study in order to determine whether changes in insulin secretion and/or insulin sensitivity can be detected early in life.

## Materials and Methods

### CASyMIR cohort

The CASyMIR cohort (**C**roissance **A**nténatale **Sy**ndrome **M**étabolique et **I**nsulino-**R**ésistance) is a French prospective cohort exploring the metabolic consequences in early infancy of being born SGA. The infants were born to women of Caucasian origin recruited during their first or second trimester of pregnancy in the maternity of the Robert Debré Hospital in Paris.

### Ethical statements

The Ethics Committee Ile-de-France 4 approved the study and written consent was obtained from both parents for all children.

### Inclusion and exclusion criteria

Inclusion criteria were the presence of one of the risk factors of delivering an SGA baby : preexisting hypertension, smoking more than five cigarettes per day, history of small for gestational age baby either in a previous pregnancy or among parents, a history of pregnancy-induced hypertensive disorder, maternal height less than 152 cm corresponding to −2SD of the mean height for French women, uterine malformations, abnormal uterine or umbilical artery Doppler and small fetal size at second trimester ultrasound examination (abdominal circumference and/or femoral length at 22 weeks of gestation). All newborns were evaluated at birth. Newborns with fetal or congenital diseases that could affect fetal growth were excluded (TORCH infections: Toxoplasmosis, Other (syphilis, varicella-zoster, parvovirus B19), Rubella, Cytomegalovirus (CMV), Herpes infections, and congenital malformation). Gestational age below 34 weeks of gestation (WG) or newborns presenting with a severe neonatal condition were also not included in the post-natal follow-up.

### Study population

Results of obstetrical, anthropometric and metabolic assessments were obtained at the age of 4 for 85 children included at birth in the CASyMIR cohort. The number of participating children was decreasing during the follow-up (at 1 year, 162 children had the anthropometric and hormonal evaluation; this number decrease to 122 children at 2 years and 104 at 3 years of life), but the data on body size and hormonal levels at birth and at age of 1 year did not differ significantly between subjects who completed the 4 year follow-up and those who did not. We could observe a difference regarding the risk factors among mothers. The children of mothers with short stature or placental vascular problems were more likely to complete the follow-up.

### Assessment of fetal growth

Fetal growth was assessed every 4 weeks by ultrasound from 22 to 36 week of gestation. All four ultrasound scans were performed by the same observer for each woman under a standardized protocol. Estimated fetal weight (EFW) was calculated using the second Hadlock formula, which includes abdominal, head circumferences, and femur length measurements [11].

One way to understand whether smallness is due to a pathological condition is the utilisation of individually adjustable, customized standards. In addition to gestational age and gender, other pregnancy characteristics, such as maternal height and weight before pregnancy, parity, and ethnicity, count for a considerable part of the variation in fetal growth velocity and weight at birth [12]. A computer program, named “Customized birth weight standards” (Gestation related optimal weight program. Software version 5.15 and Centile calculator software v5.12.1 March 2007, www.gestation.net) has been created by Gardosi et al. in which estimated fetal weight (EFW) centiles are adjusted for all these variables [13]. Using this program we were able to identify the children who reached their “optimal fetal development” and those who did not due to growth restriction.

All the babies were classified at birth as SGA or AGA. For the purpose of the study, SGA was defined as birth weight ≤−1.5 SD and AGA >−1.5 SD according to the French reference curves for gender and gestational age [14].

### Measurements

A trained midwife or pediatrician performed the clinical measurements in all children at birth. Weight was measured using an electronic scale to the nearest 10 grams. Supine length was measured to the nearest 0.5 cm with a standardized length board consisting of a fixed board for the infant's head and a movable board allowing feet to be placed perpendicular to the longitudinal axis of the infant. Body mass index (BMI = weight/length^2^) at 1and 4 years of age were calculated. Weight, length and BMI were converted into z-scores to adjust for age and sex using the French references curves [15], [16]. Skinfold thickness measurements were recorded at birth, at 1 and 4 years of age, on the left side of the body at four different sites (biceps, triceps, subscapular and suprailliacal), by the same trained pediatrician dedicated to the study. Two separate measurements were performed with a skinfold caliper (Harpenden skinfold caliper, Baty international, England) and the mean was recorded [17]. Total subcutaneous fat mass was evaluated using the sum of the four skinfold thicknesses. Percentage of body fat was derived from four skinfold measurements from the equations of Brook and Siri [18], [19].

Body composition at 4 years of age (fat mass, lean mass, and bone mineral density) was assessed by dual X-ray absorptiometry (DEXA) scan (LunaR Prodigy DXP, GE Medical Systems, Madison, WI, USA), with a specific program for small body weight [20], [21].

Blood pressure was measured on the right arm of seated subjects after 5 min of rest, using an automated device (Dinamap, Critikon, Neuilly-Plaisance, France) and a cuff of recommended size for the mid-upper arm circumference.

At 4 years of age, glucose tolerance was assessed using an oral glucose tolerance test (OGTT) performed with the administration of 1.75 g of glucose solution per kilogram of body weight, after an overnight fast. Blood samples were drawn at 0, 30 and 120 min for measurements of glucose and insulin.

### Assays

Hormonal analyses were performed at birth on a mixed venous and arterial cord blood sample. At 1 and 4 years of age, a venous sample was obtained after an overnight fast. Glucose was measured immediately whereas samples for hormonal analysis were quickly centrifuged and serum was separated and stored at −80°C until analysis. Serum insulin was measured by an IRMA kit (BI-INS-IRMA) from Cis Bio international (Gifsur-Yvette, France). Cross-reactivity with pro insulin and derived metabolites was less than 1%. Assay sensitivity was 3.0 pmol/L. Serum leptin was measured using a specific radioimmunoassay (Linco research, St Charles, USA). Sensitivity of the assay is 0.4 ng/ml. Intra- and inter- assay coefficients of variation are 5.2% and 8.7% respectively at 2.3 ng/ml.

Insulin sensitivity was assessed from fasting insulin and glucose levels using the index QUICKI (Quantitative insulin sensitivity check index) as 1/(log (fasting insulin)+log (fasting glucose) [22].

Insulinogenic index, calculated as the ratio of the increment of plasma insulin to that of plasma glucose during the first 30 min of OGTT, was used to assess beta cell function. : insulinogenic index = (insulin 30−insulin 0)/(glucose 30−glucose 0) [23], [24].

The disposition index describes the capacity of the pancreatic b-cells to secrete additional insulin to compensate over time for alterations in insulin sensitivity. It is calculated as the product of insulin secretion and sensitivity derived from OGTT (ΔI30/ΔG30×ISI).

Insulin sensitivity index (ISI Index) proposed by Matsuda and DeFronzo [25] was calculated as follows: ISI = 10,000/square root of [fasting glucose×fasting insulin]×[mean glucose×mean insulin during OGTT]

### Statistical analysis

Statistical analyses were performed using the SAS software version 9.1.3 for Windows (SAS statistical package, SAS institute, Meylan France).

Data are given as mean ± SD. Univariate analyses were performed for the comparison between the 2 groups using the Chi-2 test for qualitative variables and the Student's t-test, or nonparametric tests when appropriate, for quantitative variables.

For hormonal parameters, the comparisons between 2 groups of newborns were made by using general linear model with fat mass as covariate.

## Results

**Figure 1:**
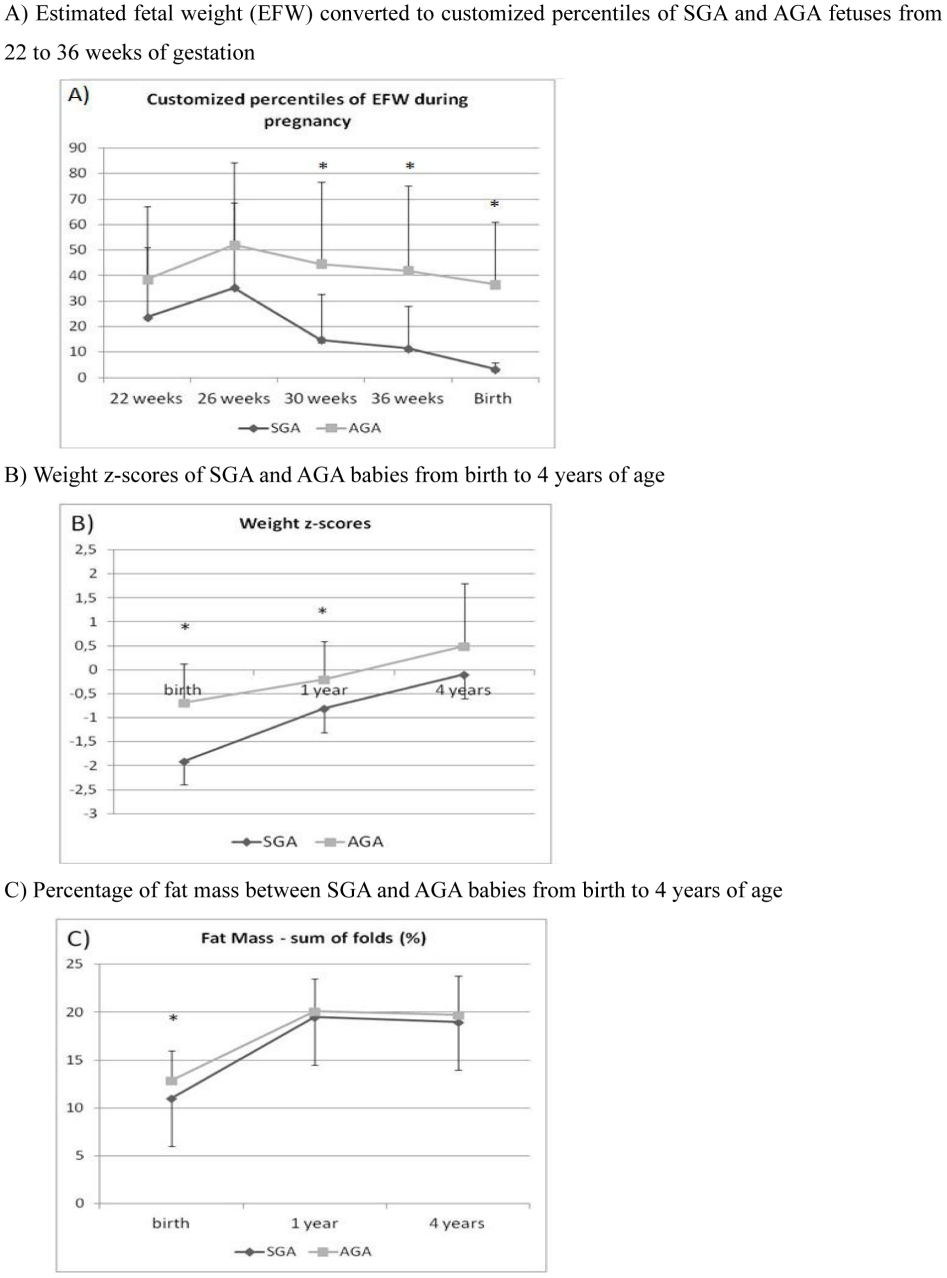
A) Estimated fetal weight (EFW) converted to customized percentiles of SGA and AGA fetuses from 22 to 36 weeks of gestation. B) Birth weight z-scores of SGA and AGA babies from birth to 4 years of age. C) Percentage of fat mass between SGA and AGA babies from birth to 4 years of age.

The anthropometric and hormonal characteristics between groups SGA and AGA children at birth, 1 and 4 years are given in Table 1. Figure 1A shows fetal weight changes from 22 weeks of gestation to birth. From 30 weeks of gestation, EFW was significantly lower in SGA subjects (14.8±17.9 vs. 44.6±32.2 percentiles, p<0.0001 at 30 weeks of gestation; 11.4±16.6 vs. 42.7±33.4 percentiles, p<0.0001 at 36 weeks of gestation) showing that SGA babies slowed down fetal growth during the 3^rd^ trimester of pregnancy.

**Table 1:** Estimated fetal weight (EFW) from 22 weeks of pregnancy until birth and anthropometric and hormonal characteristics at birth, 1 and 4 years of life between SGA and AGA babies.

Variable	SGA	AGA	p
	n = 23	n = 62	
**Percentiles of EFW**			
22 weeks of gestation	23.7±27.4	38.6±28.6	0.05
26 weeks of gestation	35.3±33.2	52±32.4	0.08
30 weeks of gestation	14.8±17.9	44.6±32.2	***<0.0001***
36 weeks of gestation	11.4±16.6	42.7±33.4	***<0.0001***
Birth percetiles	3.3±2.6	36.6±24.5	***<0.0001***
**Birth**			
Female gender (%)	13 (56.5)	34 (54.8)	0.89
Gestational age (wk)	38.8±2	38.9±1.7	0.8
Birth weight (g)	2363.3±384.3	2929.5±507.6	***<0.0001***
Birth weight (z-score)	−1.9±0.3	−0.6±0.8	***<0.0001***
Birth Lenght (cm)	45.5±2.9	48±2.3	***<0.0001***
Birth Lenght (z-score)	−1.9±0.8	−0.6±1	***<0.0001***
Cranial perimeter (cm)	32.6±1.7	33.8±1.6	***0.006***
Ponderal index (kg/m3)	24.8±1.8	26.3±3.1	***0.01***
Sum of folds (mm)	14.2±2.3	16.7±3.5	***0.001***
Fat Mass - sum of folds (%)	11±2.6	12.9±3.1	***0.01***
Glucose (mmol/l)	4.7±1.1	4.5±1.1	0.9
Triglyceride (mg/dl)	0.69±0.4	0.5±0.2	0.22
Cord insulin (mUI/l)	4.1±3.5	4.5±5.3	0.87
Cord leptin (ng/ml)	5.5±6.3	6.9±8.2	0.47
QUICKI	0.48±0.1	0.47±0.1	0.89
**1 year of age**			
Weight (kg)	8.7±6	9.3±0.9	***0.0006***
Weight (z-score)	−0.8±0.6	−0.2±0.8	***0.0005***
Height (cm)	72.7±1.9	73.9±2.3	***0.02***
Height (z-score)	−0.4±0.7	0.08±0.9	***0.02***
BMI (kg/m^2^)	16.4±1.1	17±1.1	***0.04***
BMI (z-score)	−0.7±0.9	−0.2±0.8	***0.04***
Cranial perimeter (cm)	45.7±1.7	45.9±1.5	0.57
Delta weight 1 yr-birth	1.1±0.6	0.4±0.9	***0.002***
Sum of folds (mm)	28.8±5	30.5±6.5	0.27
Fat Mass - sum of folds (%)	19.5±3.3	20.1±3.4	0.49
Glucose (mmol/l)	4.4±0.5	4.6±0.4	0.14
Insulin (mUI/l)	2.1±1.8	2.3±2.3	0.75
Leptin (ng/ml)	2.9±1	3.7±1.9	0.05
QUICKI	0.51±0.1	0.54±0.2	0.38
**4 years of age**			
Weight (kg)	15.2±1.8	16.3±1.9	***0.02***
Weight (z-score)	−0.1±1.3	0.5±1.3	***0.05***
Height (cm)	100.3±3.1	102.1±3.6	***0.03***
Height (z-score)	0.4±1	0.8±1.1	0.1
BMI (kg/m^2^)	15.1±1.3	15.6±1.2	0.08
BMI (z-score)	−0.4±1.1	0.01±1	0.08
Cranial perimeter (cm)	50±1.5	50.3±1.7	0.26
Sum of folds (mm)	28.3±9.2	30±7.3	0.40
Fat Mass - sum of folds (%)	19±4.8	19.7±4.1	0.45
Systolic BP (mmHg)	101.4±10.8	100.4±10.6	0.71
Diastolic BP(mmHg)	51±10.5	55.3±12.7	0.14
Lean Mass (g)	11741.8±932.1	12319.2±1889.2	0.09
Fat Mass (g)	2519.6±1338.2	2723.4±1075	0.5
Bone mineral content (g/cm)	419.2±58	512.4±79.3	0.21
Triglyceride (mg/dl)	0.6±0.1	0.6±0.2	0.95
Total cholesterol (mmol/l)	4.1±0.6	4.1±0.8	0.94
HDL cholesterol (mmol/l)	41.3±24.6	35.1±25	0.36
Leptin (ng/ml)	4.3±3.8	3.8±1.8	0.22
Glucose t0 (mmol/l)	4.2±0.8	4.6±0.5	***0.01***
Glucose t30 (mmol/l)	7.9±1.6	8.3±1.8	0.38
Glucose t120 (mmol/l)	6.2±1.1	5.6±0.9	***0.006***
Insulin t0 (mUI/l)	2.4±1.5	3±1.7	0.2
Insulin t30 (mUI/l)	21±15.1	28.1±16.2	0.11
Insulin t120 (mUI/l)	10.2±5.3	9.1±5.1	0.36
QUICKI	0.47±0.09	0.43±0.05	0.06
Insulinogenic index	0.28±0.15	0.40±0.24	***0.02***
Disposition Index	8.3±5.3	10.2±6.3	0.29

The SGA babies were thinner at birth with significantly lower ponderal index (24.8±1.8 vs. 26.3±3.1 kg/m3; p = 0.01) and lower total subcutaneous fat mass assessed by the sum of skin folds (11±2.6 vs. 12.9±3.1%; p = 0.01). No other significant differences in any of the measured metabolic and hormonal parameters were observed between SGA and AGA subjects at birth.

The time-course of weight (expressed in z-scores - Figure 1B) illustrates that only SGA infants had experienced an early catch-up growth with a mean gain of 1.1±0.6 SD during the first year of life, correcting for the loss seen at birth. Height z-scores followed the same pattern of catch-up growth so that, although the mean height z-score was significantly lower at 1 yr of age in SGA, none of the SGA children presented with a short stature (height z-score < −2SD) at that age. Catch-up growth was not associated with excessive fat mass measured by the sum of skin folds (Figure 1C). Accordingly, leptin levels were lower in SGA children (2.9±1vs. 3.7±1.9; p = 0.05). By contrast, there was no difference in any other measured metabolic and hormonal parameters at 1 year of age.

**Figure 2:**
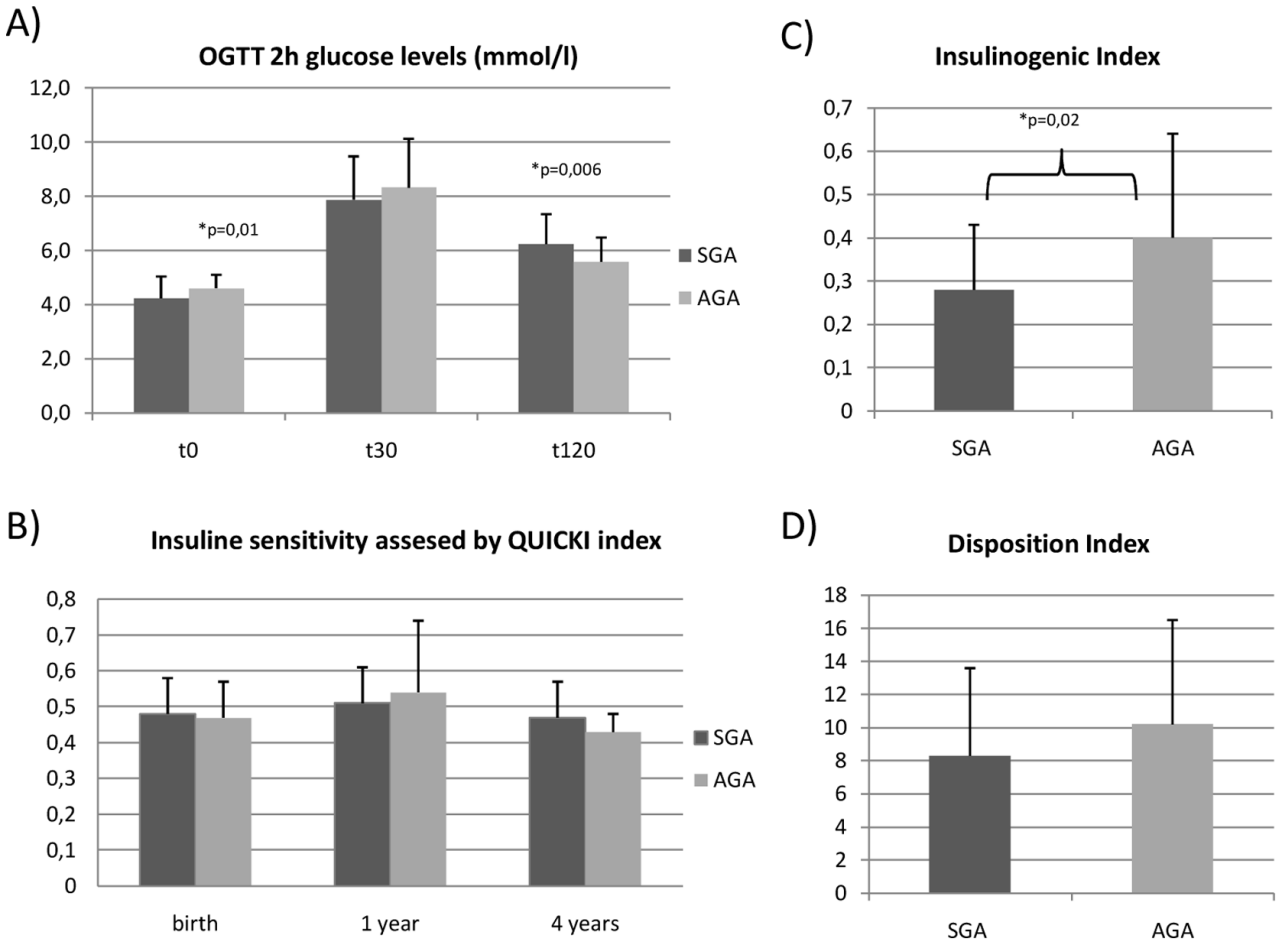
A) Changes in glucose levels during the OGTT between SGA and AGA children at 4 years of age. B) Changes in insulin sensitivity assessed by Quicki Index from birth to 4 years of age between SGA and AGA children. C) Insulinogenic index values between SGA and AGA children at 4 years of age. D) Disposition index values between SGA and AGA children at 4 years of age.

At 4 years of age, even if SGA children were somewhat lighter than AGA children, weight z-score were in the normal range (−0.1±1.3 vs. 0.5±1.3 z-score; p = 0.05) and no significant difference in fat mass measured by either the sum of skin fold or by DEXA, was observed. Lean mass was also similar between SGA and AGA children at the age of 4 years (11741.8±932.1 vs. 12319.2±1889.2; p = 0.09). Surprisingly, OGTT data attested for mild glucose tolerance disorders in the SGA group: indeed, plasma glucose 2 hours after the oral glucose load was significantly higher in the SGA group (6.2±1.1 vs. 5.6±0.9 mmol/l; p = 0.006) despite lower fasting plasma glucose (4.2±0.8 vs. 4.6±0.5 mmol/l; p = 0.02) (Figure 2A). Insulinogenic index was significantly lower in the SGA group than in the AGA group (0.28±0.15 vs. 0.40±2.4; p = 0.02) at 4 years of life (Figure 2C) contrasting with a preserved insulin sensitivity (QUICKI 0.47±0.09 vs. 0.43±0.05; p = 0.06) (Figure 2B).

No significant difference in lipid results was observed between two groups of children at the age of 4.

To note, calorie intake was similar in both groups at all ages (data not shown). In our study, at the age of four months (data not shown) we observed a difference in the type of feeding between the two groups of children with the AGA children that were more likely to be exclusively breast fed. But weight gain was still significantly different between children born small and those born with a normal weight, even after adjustment for type of feeding.

No type 1 or type 2 diabetes was found on the mother's side in the two groups of children. On the father's side one case (4.3%) of type 1 diabetes was noted in the SGA group, and one of type 2 diabetes in each group of children (data not shown).

## Discussion

Our study is the first prospective one that studied insulin secretion in young healthy children born SGA in relationship with fetal and postnatal growth and associated changes in body composition. We found that SGA children do not have changes in insulin sensitivity but have lower glucose stimulated insulin secretion by comparison with AGA children at the age of four.

Furthermore, we found that catch-up growth following fetal growth restriction does not induce changes in insulin resistance or fat mass excess. The lack of these confounding factors that can affect insulin sensitivity and then increase fasting and stimulated insulin secretion allowed us to show that SGA children had defect in stimulated insulin secretion, suggesting that the exposure to an adverse nutritional environment during fetal life may affect pancreatic β-cell development, leading to impaired β-cell function later in the life.

Numerous animal studies already pointed that fetal growth restriction alters the endocrine function of the pancreas. For example, in sheep, chronic restriction of nutrient supply due to poor placental growth induces an intrauterine growth restriction (IUGR) associated to a reduced β-cell replication, and consequently, a reduced β-cell mass. Such changes were associated with changes in beta cell genes expression and then to a decreased pancreatic insulin content which reduced the capacity for insulin secretion [26]. The same defects were observed in fetal growth restricted rats, which also developed significant β-cell hypoplasia [27]–[29].

Human studies mostly reported that children with restricted fetal growth leading to thinness at birth were these who experienced catch up growth during the first months of life. Numerous clinical data also suggested that catch-up growth was a risk factor of developing excessive central fat mass and insulin resistance, both contributing to favor the development of metabolic diseases later in life [30]–[35]. Few studies reported an impaired insulin secretion in SGA subjects and mostly in adults [36]. Cook and al. [37] reported a reduced beta cell function in a small number of adult subjects born with low birth weight and having a family history of type 2 diabetes. One may ask whether this decrease was explained by the birth weight or by genetic factors. In our study, no family history of type 1 or type 2 diabetes was found in SGA children, so the lower glucose stimulated insulin secretion can be a consequence of intra uterine growth restriction and of consecutively impaired β-cell function.

Ong et al. examined associations between size at birth, postnatal weight gain, circulating IGF-I levels and insulin sensitivity and secretion in 8 years old children. They found that the association between low birth weight and insulin resistance may be dependent on rapid weight gain during the early postnatal years. But, irrespective of postnatal weight gain, smaller size at birth, lower IGF-I levels and lower childhood height predicted reduced compensatory insulin secretion [38].

Mericq and al. [39] studied insulin secretion and sensitivity at the age of 3 years in SGA and AGA children. They found that the development of insulin resistance occurred in early postnatal life in case of rapid catch-up growth. Moreover, they observed a reduced compensatory beta cell secretion independent of postnatal catch-up growth.

In the present study, SGA children did not show excessive weight gain, neither changes in fat mass nor insulin resistance at the age of 4. We previously reported that early catch-up growth following fetal growth restriction promoted restoration of fat storage but did not induce excess of fat mass or unfavorable changes either in body composition or in insulin sensitivity at one year of age. This catch-up growth was then an adaptive and “physiological” phenomenon [40]. So, we could conclude that catch-up growth was not deleterious for infants and was an adaptive phenomenon to compensate for fetal growth restriction. By the contrast, an excessive one could promote an excess of fat mass and the appearance of IR and then the risk of type 2 diabetes later in the life.

In our study catch-up growth was symmetrical in weight and height (even slightly more important in height) and independent of postnatal feeding or later caloric intake explaining the lack of IR or excess in fat mass at the age of 4. In case of asymmetric catch-up growth, we could think that the insulin resistance is rather a consequence of excessive postnatal weight gain then of the catch-up growth itself.

In the present study, subjects born SGA showed a reduction in early phase insulin secretion with normal insulin levels in the late phase. Because early phase insulin secretion is important in priming the liver and inhibiting endogenous glucose production during OGTT [41], this defect may reasonably account for the highest glucose levels we observed in OGTT late values in the SGA group.

Children in our study did not show any difference in insulin sensitivity but they had a lower glucose stimulated insulin secretion at 4 years of age. We calculated the Disposition index in order to characterize β-cell function according to the insulin sensitivity and we founded it to be lower in the SGA group but without statistically significant difference. We believe that this absence of significance is more reflecting the lack of statistical power then a real lack of difference.

Changes in pancreatic function seem dependent on the model and timing of fetal growth restriction. Early gestation was identified long time ago as a critical window. In a recent Indian study, the authors did not find an effect of maternal under nutrition in mid-pregnancy pregnancy on human fetal pancreas morphology [42]. By the contrast, in our study, SGA children experienced fetal growth restriction in the 3^rd^ trimester of pregnancy and were thinner at birth, so we believe that they were not “constitutionally small babies” and we can argue for an effect of fetal growth restriction during that moment of pregnancy on pancreas development. The time of beginning of follow-up (22 weeks) did not allow us to know if fetal growth restriction also occurred in the early pregnancy.

Some limits must be taken into account in our study. First, we did not study insulin sensitivity with the gold standard of the euglycemic hyperinsulinemic clamp. Indeed this is a rather complicated method that involves the hospitalization and continuous intravenous administration of insulin and glucose over a period of 3 hours. The use of euglycemic clamp would have been complicated in such young children and ethically doubtable as most of the children in our study were healthy. Another limit of our study is the lack of an independent control group i.e children without risk factors of being born SGA. This kind of comparison would have strengthened our results.

All longitudinal studies are subject to selection bias caused by difficulties to include and keep the children until the end of the follow-up. This raises the question of lack of statistical power of the study, and demands a careful interpretation of our results and confirmation studies. The small number of subjects in our case can be explained by the fact that children were followed prospectively including the fetal period and measurement of fetal growth. Such cohorts are still exceptional and very difficult to organize.

In conclusion, healthy SGA children that experience a compensatory catch-up growth, leading to the restoration of their body composition at the age of four without relative fat mass excess, show mild disturbances of glucose tolerance associated to a lower glucose stimulated insulin secretion at 4-yrs of age suggesting impairment of β-cell function in line with the already described predisposition to type 2 diabetes later in life.
