# Correction: SGA Children with Moderate Catch-Up Growth Are Showing the Impaired Insulin Secretion at the Age of 4

**DOI:** 10.1371/journal.pone.0108008

**Published:** 2014-09-16

**Authors:** The PLOS ONE Staff

The last 17 rows of Table 1 do not appear. Please see the complete Table 1 here.

**Table 1:** Estimated fetal weight (EFW) from 22 weeks of pregnancy until birth and anthropometric and hormonal characteristics at birth, 1 and 4 years of life between SGA and AGA babies.

Variable	SGA	AGA	p
	n = 23	n = 62	
**Percentiles of EFW**			
22 weeks of gestation	23.7±27.4	38.6±28.6	0.05
26 weeks of gestation	35.3±33.2	52±32.4	0.08
30 weeks of gestation	14.8±17.9	44.6±32.2	***<0.0001***
36 weeks of gestation	11.4±16.6	42.7±33.4	***<0.0001***
Birth percetiles	3.3±2.6	36.6±24.5	***<0.0001***
**Birth**			
Female gender (%)	13 (56.5)	34 (54.8)	0.89
Gestational age (wk)	38.8±2	38.9±1.7	0.8
Birth weight (g)	2363.3±384.3	2929.5±507.6	***<0.0001***
Birth weight (z-score)	−1.9±0.3	−0.6±0.8	***<0.0001***
Birth Lenght (cm)	45.5±2.9	48±2.3	***<0.0001***
Birth Lenght (z-score)	−1.9±0.8	−0.6 ±1	***<0.0001***
Cranial perimeter (cm)	32.6±1.7	33.8±1.6	***0.006***
Ponderal index (kg/m3)	24.8±1.8	26.3±3.1	***0.01***
Sum of folds (mm)	14.2±2.3	16.7±3.5	***0.001***
Fat Mass - sum of folds (%)	11±2.6	12.9±3.1	***0.01***
Glucose (mmol/l)	4.7±1.1	4.5±1.1	0.9
Triglyceride (mg/dl)	0.69±0.4	0.5±0.2	0.22
Cord insulin (mUI/l)	4.1±3.5	4.5±5.3	0.87
Cord leptin (ng/ml)	5.5±6.3	6.9±8.2	0.47
QUICKI	0.48±0.1	0.47±0.1	0.89
**1 year of age**			
Weight (kg)	8.7±6	9.3±0.9	***0.0006***
Weight (z-score)	−0.8±0.6	−0.2±0.8	***0.0005***
Height (cm)	72.7±1.9	73.9±2.3	***0.02***
Height (z-score)	−0.4±0.7	0.08±0.9	***0.02***
BMI (kg/m^2^)	16.4±1.1	17±1.1	***0.04***
BMI (z-score)	−0.7±0.9	−0.2±0.8	***0.04***
Cranial perimeter (cm)	45.7±1.7	45.9±1.5	0.57
Delta weight 1yr-birth	1.1±0.6	0.4±0.9	***0.002***
Sum of folds (mm)	28.8±5	30.5±6.5	0.27
Fat Mass - sum of folds (%)	19.5±3.3	20.1±3.4	0.49
Glucose (mmol/l)	4.4±0.5	4.6±0.4	0.14
Insulin (mUI/l)	2.1±1.8	2.3±2.3	0.75
Leptin (ng/ml)	2.9±1	3.7±1.9	0.05
QUICKI	0.51±0.1	0.54±0.2	0.38
**4 years of age**			
Weight (kg)	15.2±1.8	16.3±1.9	***0.02***
Weight (z-score)	−0.1±1.3	0.5±1.3	***0.05***
Height (cm)	100.3±3.1	102.1±3.6	***0.03***
Height (z-score)	0.4±1	0.8 ±1.1	0.1
BMI (kg/m^2^)	15.1±1.3	15.6±1.2	0.08
BMI (z-score)	−0.4±1.1	0.01±1	0.08
Cranial perimeter (cm)	50±1.5	50.3±1.7	0.26
Sum of folds (mm)	28.3±9.2	30±7.3	0.40
Fat Mass - sum of folds (%)	19±4.8	19.7±4.1	0.45
Systolic BP (mmHg)	101.4±10.8	100.4±10.6	0.71
Diastolic BP(mmHg)	51±10.5	55.3±12.7	0.14
Lean Mass (g)	11741.8±932.1	12319.2±1889.2	0.09
Fat Mass (g)	2519.6±1338.2	2723.4±1075	0.5
Bone mineral content (g/cm)	419.2±58	512.4±79.3	0.21
Triglyceride (mg/dl)	0.6±0.1	0.6±0.2	0.95
Total cholesterol (mmol/l)	4.1±0.6	4.1±0.8	0.94
HDL cholesterol (mmol/l)	41.3±24.6	35.1±25	0.36
Leptin (ng/ml)	4.3±3.8	3.8±1.8	0.22
Glucose t0 (mmol/l)	4.2±0.8	4.6±0.5	***0.01***
Glucose t30 (mmol/l)	7.9±1.6	8.3±1.8	0.38
Glucose t120 (mmol/l)	6.2±1.1	5.6±0.9	***0.006***
Insulin t0 (mUI/l)	2.4±1.5	3±1.7	0.2
Insulin t30 (mUI/l)	21±15.1	28.1±16.2	0.11
Insulin t120 (mUI/l)	10.2±5.3	9.1±5.1	0.36
QUICKI	0.47±0.09	0.43±0.05	0.06
Insulinogenic index	0.28±0.15	0.40±0.24	*0.02*
Disposition Index	8.3±5.3	10.2±6.3	0.29

## References

[1] Milovanovic I, Njuieyon F, Deghmoun S, Chevenne D, Levy-Marchal C, et al (2014) SGA Children with Moderate Catch-Up Growth Are Showing the Impaired Insulin Secretion at the Age of 4. PLoS ONE 9(6): e100337 doi:10.1371/journal.pone.0100337 24979613 10.1371/journal.pone.0100337 PMC4076235

